# Role of Astaxanthin as a Stimulator of Ovarian Development in Nile Tilapia (*Oreochromis niloticus*) and Its Potential Regulatory Mechanism: Ameliorating Oxidative Stress and Apoptosis

**DOI:** 10.1155/2022/1245151

**Published:** 2022-09-10

**Authors:** Jun Qiang, Yi-Fan Tao, Si-Qi Lu, Jun-Lei Ma, Jie He, Pao Xu

**Affiliations:** ^1^Key Laboratory of Freshwater Fisheries and Germplasm Resources Utilization, Ministry of Agriculture, Freshwater Fisheries Research Center, Chinese Academy of Fishery Sciences, Wuxi, 214081 Jiangsu, China; ^2^Wuxi Fisheries College, Nanjing Agricultural University, Wuxi 214081, China

## Abstract

A 60-day feeding experiment was performed to evaluate the effect of dietary astaxanthin on gonad development, the antioxidant system, and its inherent mechanism in female Nile tilapia (*Oreochromis niloticus*). Fish were fed with diets containing astaxanthin at five levels [0 mg/kg (control), 50 mg/kg, 100 mg/kg, 150 mg/kg, and 200 mg/kg]. At the end of experiment, the group fed with 150 mg/kg astaxanthin showed significantly increased specific growth rate, feed utilization, viscerosomatic index, and hepatosomatic index compared with the control group (*P* < 0.05). Gonad development was stimulated in the groups fed with 100 mg/kg and 150 mg/kg astaxanthin, and their gonadosomatic index and egg diameter were significantly higher than those of the control group and the group fed with 200 mg/kg astaxanthin. The ovaries of females in the groups fed with 100 mg/kg and 150 mg/kg astaxanthin were fully developed, the eggs were gray-yellow and uniform in size, and a large number of oocytes developed to stages IV and V. The serum levels of 17 *β*-estradiol, follicle-stimulating hormone, and luteinizing hormone were significantly higher in the groups fed with 100 mg/kg and 150 mg/kg astaxanthin than in the group fed with 200 mg/kg astaxanthin. Compared with the control and the other groups, the group fed with 150 mg/kg astaxanthin showed significantly higher transcript levels of genes encoding hormone receptors and higher catalase activity in ovarian tissues, lower malondialdehyde content, decreased apoptosis (reduced granulosa cell apoptosis and lower transcript levels of *bax* and *caspase-3*), and reduced follicular atresia. Gene ontology analyses revealed that cell division and the cell cycle were enriched with differentially expressed genes in the group fed with 150 mg/kg astaxanthin, compared with the control group. We concluded that dietary astaxanthin at a concentration of 150 mg/kg activates follicle development by inhibiting expression of *mapk1* (involved in MAPK signaling) and increasing the expression genes involved in oocyte meiosis (*chp2*, *ppp3ca*, *map2k1*, and *smc1a1*) and progesterone-mediated oocyte maturation (*igf1*, *plk1*, and *cdk1*). In conclusion, female Nile tilapia fed with 150 mg/kg astaxanthin showed increased growth, reduced oxidative stress in ovarian tissue, lower levels of cell apoptosis, and improved oocyte development.

## 1. Introduction

Astaxanthin is a small molecule with unsaturated hydroxyl and ketone groups [1]. It was the only pigment additive allowed in the feed additive catalog [2]. Astaxanthin can combine with myoglobin in the body to make fish skin brilliantly colored, and it regulates the deposition of body pigment [3]. Many studies have shown that dietary supplementation with astaxanthin affects the pigmentation and nonspecific immune function of fish [2, 4–6]. Atlantic salmon (*Salmo salar*) fed with a diet containing 88.6 mg/kg astaxanthin accumulated astaxanthin in the muscle to a concentration of 2.0–2.5 *μ*g/kg, compared with 0.5–0.8 *μ*g/kg in the control group [6]. Astaxanthin-supplemented feeds were shown to promote the growth, immunity, and body color of oscar (*Astronotus ocellatus*) [2] and increase the growth of common carp (*Cyprinus carpio*) and its resistance to *Aeromonas hydrophila* [5]. Lobster meal rich in astaxanthin and protein-supplemented feeds were shown to increase the brightness, redness, and yellowness of goldfish (*Carassius auratus*) [4]. Because the molecular structure of astaxanthin contains conjugated double bonds as well as hydroxyl and ketone groups and other reducing groups, it can react with oxygen free radicals to scavenge free radicals. Thus, it has powerful antioxidant effects [1]. The addition of astaxanthin to the diet of large yellow croaker (*Larimichthys crocea*) was shown to increase the activity of hepatic superoxide dismutase and glutathione peroxidase, thereby increasing the antioxidant capacity [7].

Gonad development and the timing of sexual maturity in broodstock, the fertilization rate, the hatching rate of fertilized eggs, and the survival and quality of fry are affected by the nutritional status of the feed (i.e., the types and proportions of protein and amino acids, fat and fatty acids, vitamins, minerals, and other nutrients) [8, 9]. Reproductive performance can be improved by appropriate nutrition but reduced by overnutrition or undernutrition [10]. Poor nutrition can even result in death. In recent years, astaxanthin has attracted attention because it can improve the reproduction level of female broodstock. Astaxanthin-supplemented feeds have been shown to increase the spawning rate of striped jack (*Pseudocaranx dentex*) [11] and the fertilization rate, egg survival rate, and growth of freshwater crayfish (*Astacus leptodactylus*), black tiger shrimp (*Penaeus monodon*), and Atlantic cod (*Gadus morhua* L.) [12–14]. Adding krill meal as the main protein source to the feed of gilthead bream (*Sparus aurata*) was shown to significantly improve the reproductive performance of the broodstock [9], because krill meal contains phosphatidylcholine and astaxanthin, which are required for gonad development [9].

Aquatic germplasm resources are essential for the development of modern aquaculture, and the demand for high-quality fry has increased significantly as the aquaculture industry has developed. Tilapia, as an important imported species in China, has experienced serious germplasm degradation in recent years and has shown slowed growth, decreased fecundity, and increased susceptibility to disease [15, 16]. Therefore, it is very important to investigate how various nutrients can improve the reproductive ability of tilapia broodstock and the quality of offspring. In this study, we explored the effect of astaxanthin-supplemented feeds on the reproductive ability of Nile tilapia (*Oreochromis niloticus*) and the potential mechanism of this effect. The results provide a scientific basis for promoting the reproduction of tilapia through nutritional means.

## 2. Materials and Methods

### 2.1. Experimental Fish

One-year-old experimental female fish were obtained from the Yixing Base of the Freshwater Fisheries Research Center of the Chinese Academy of Fishery Sciences (FFRC). The experimental fish were initially kept in an indoor temperature-controlled (water temperature 28 ± 0.5°C) circulating water system for 15 days. During the acclimation period, the dissolved oxygen (DO) in the water was measured daily and maintained at (DO) >7.09 mg/L. The ammonia nitrogen concentration was lower than 0.5 mg/L, and the pH was 7.3 ± 0.2. Fish were fed with commercial feed (33% protein and 6.5% lipid) at 08 : 00 and 16 : 00 at 5% of body weight.

### 2.2. Preparation of Experimental Feed

We formulated five experimental diets without or with astaxanthin at different concentrations [0 mg/kg (control), 50 mg/kg, 100 mg/kg, 150 mg/kg, and 200 mg/kg] (Table 1). We used natural astaxanthin derived from *Haematococcus pluvialis* (purity 10%), which was purchased from Shangcheng Biotechnology Co., Ltd. (Xi'an, China). Fish meal, soybean meal, and rapeseed meal were used as protein sources. Soybean oil was the lipid source. Wheat flour and corn flour were used as carbohydrate sources. All the diets were energetically equal. The dry materials were mixed to homogeneity in a Hobart mixer at the FFRC, and then the wet materials were added, and the mixture was shaped into cold-extruded pellets (2.5 mm diameter). After drying, the feeds were sealed in vacuum-packed bags and kept at −20°C until the start of the experiment. The astaxanthin content was determined by liquid chromatography [17]. A Luna 3u silica column (150 mm × 4.60 mm; Phenomenex, Torrance, CA, USA) was used for these analyses. The mobile phase was n-hexane and acetone (83 : 17, v/v; flow rate 1.0 mL/min). The detection wavelength was 478 nm, and the injection volume was 20 *μ*L.

### 2.3. Experimental Design and Feeding Management

All the fish were starved for 24 h at the start of the experiment, and the average initial weight was 207.5 ± 4.3 g. Then, 200 selected Nile tilapia females with well-developed gonads were randomly divided into five groups (four replicates per group and 10 fish per replicate). There was no significant variation in initial body weight among the fish groups at the start of the experiment. The rearing experiment was carried out indoors in polyethylene tanks (diameter × height = 2080 mm × 1200 mm) with circulating water systems. The females were fed by hand at 08 : 00–09 : 00 and 16 : 00–17 : 00 at the rate of 3%–5% of fish body weight. The amount of the diet consumed was recorded daily, and the feeding rate was adjusted every 15 d by determining the total weight of the fish in each tank. The DO, temperature, and pH of the water were measured every day and maintained at 6.5 ± 0.5 mg/L, 28 ± 0.5°C, and 7.53 ± 0.2, respectively. The entire experiment lasted for 60 days.

### 2.4. Sample Collection

At the end of experiment, the total weight of all experiment fish in each tank was measured. All fish were starved for 24 h to allow the alimentary tract to clear before sampling. Four fish from each tank were randomly selected for the collection of blood and gonad samples. The fish were anesthetized with an overdose of tricaine sulfonate at 200 mg/L (MS-222, Argent Chemical Laboratories, Redmond, WA, USA). Blood samples were collected from four fish per tank using a 2.5-mL syringe and were placed into 1.5-mL Eppendorf tubes. The blood samples were centrifuged at 5000 g for 15 min at 4°C. The supernatant (serum) was stored at −80°C until analysis. After the fish were weighed, the ovarian tissue was quickly dissected and weighed. A portion of gonadal tissue was taken from the anterior, middle, and posterior parts of the ovary and fixed in Bouin's solution, fixed for 24 h, and then stored in 70% v/v ethanol. Gonad tissues from another four experimental fish from each tank were instantly frozen in liquid nitrogen and kept at -80°C until transcriptome and gene expression analysis.

### 2.5. Parameter Measurement

#### 2.5.1. Growth Performance

Growth parameters were calculated according to the following equations:

(1) $$\begin{array}{c} \text{Weight gain}\left(\text{WG},\%\right)=\frac{\text{final bodyweight}\left(\text{FBW},\text{g}\right)–\text{initial bodyweight}\left(\text{IBW},\text{g}\right)}{\text{IBW}\left(\text{g}\right)}\times 100,\\ \text{Specific growth rate}\left(\text{SGR},\%/\text{day}\right)=\frac{\left[\text{lnFBW}\left(\text{g}\right)–\text{lnIBW}\left(\text{g}\right)\right]}{\text{number of days}}\times 100,\\ \text{Feed conversion ratio}\left(\text{FCR}\right)=\frac{\text{fish feed intake}}{\text{FBW}–\text{IBW}},\\ \text{Hepatosomatic index}\left(\text{HSI},\%\right)=\frac{\text{liver mass}\left(\text{g}\right)}{\text{FBW}\left(\text{g}\right)}\times 100,\\ \text{Visceromatic index}\left(\text{VSI},\%\right)=\frac{\text{visceral mass}\left(\text{g}\right)}{\text{FBW}\left(\text{g}\right)}\times 100. \end{array}$$

#### 2.5.2. Gonad Development

The gonadosomatic index (GSI, %) was calculated as follows: GSI = gonad mass (g)/FBW (g) ;

For each individual female, the egg diameter was determined as the average of 100 eggs and was measured to the nearest 0.01 mm under a calibrated binocular microscope. Because tilapia eggs are ellipsoidal, we used 10 eggs as a group and measured both axes (long and short) to calculate mean egg size (mm), as follows: [∑10 eggs (length + width)/20] [18].

The relative fecundity (*F*_*R*_, eggs kg^−1^ body weight) was calculated as follows: *F*_*R*_ = 100 × total number of eggs/FBW.

#### 2.5.3. Oocyte Development and Apoptosis

*(1) Hematoxylin/Eosin (HE) Staining*. The fixed ovary sample was dehydrated, cleared, embedded in paraffin, and serially cut into 5–6*-μ*m sections. After HE staining, each section was observed and photographed using the NIKON digital sight DS-FI2 imaging system of a NIKON Eclipse Ci microscope (NIKON, Tokyo, Japan). There were six digital fields in each slice, and atretic follicles were counted in each digital field separately. The morphological analysis of follicular atresia (FA) has been described in detail elsewhere [15].

*(2) Terminal dUTP Nick-End Labeling (TUNEL) Analyses*. Sections of ovary tissues were prepared as described above. Then, DNase-free proteinase K (20 *μ*g/mL) was added dropwise to the deparaffinized and rehydrated sections, and then the sections were incubated in a humid chamber at 37°C for 30 min before adding 50 *μ*L prepared TUNEL reaction solution dropwise to the tissue in the dark. The sections were again incubated at in a humid chamber at 37°C for 60 min. After mounting the slide with antifluorescence quenching mounting solution, sections were observed and photographed under a NIKON Eclipse Ci microscope equipped with a NIKON DS-U3 imaging system. The excitation and emission wavelengths of the dyes were as follows: 4,6-diamidino-2-phenylindole (DAPI), 330–380 nm and 420 nm, respectively; fluorescein isothiocyanate (FITC), 465–495 nm and 515–555 nm, respectively. The TUNEL Apoptosis Assay Kit was purchased from Yanjin Biotechnology Co., Ltd. (Shanghai, China). The nuclei stained by DAPI were blue under ultraviolet excitation, and the nuclei of the apoptotic cells labeled with FITC were green. There were six digital fields in each slice, and the number of apoptotic cells were counted in each digital field separately.

#### 2.5.4. Antioxidant Enzyme Activity

Each thawed gonad sample was rinsed with precooled physiological saline and blotted dry on filter paper. Then, 0.1 g of the sample was homogenized with nine volumes of precooled phosphate-buffered saline (PBS). The homogenate was used to determine malondialdehyde (MDA) content and the activities of superoxide dismutase (SOD) and catalase (CAT). All indicators were measured within 24 h, and the absorbance of solutions was determined using a microplate reader (BioTek Epoch, Winooski, VT, USA). The protein concentration in the supernatant was determined by the Coomassie Brilliant Blue Assay. All experimental kits were purchased from the Enzyme-linked Biotechnology Co., Ltd. (Shanghai, China).

#### 2.5.5. Serum Hormone Levels

The follicle-stimulating hormone (FSH) and luteinizing hormone (LH) contents in serum were determined by specific and homologous competitive enzyme-linked immunosorbent assay (ELISA) methods [19]. The kits contained a microtiter plate coated with the corresponding hormone antibody (labeled with horseradish peroxide (HRP)). After the serum hormone combined with the antibody in the microtiter plate, color was developed using a development solution and HRP, and then absorbance at 450 nm was measured using a microplate reader. The hormone levels in the sample were calculated from a standard curve following the manufacturer's instructions. The same principle was used to determine the serum contents of estradiol (E_2_).

### 2.6. Library Construction and Transcriptome Sequencing

The control group and the optimal astaxanthin-supplemented group were used to construct libraries for transcriptome sequencing. The ovarian tissues from 12 fish from each group stored at −80°C were thawed in an ice box before extracting total RNA with TRIzol reagent. The quantity and purity of the total RNA were determined using a Bioanalyzer 2100 and an RNA 6000 Nano LabChip Kit (Agilent, CA, Palo Alto, USA) (RIN number> 7.0). For each group, the RNA from four samples was mixed to construct a sequencing library, with three replicates. Thus, a total of six sequencing libraries were constructed: three from the control group (Con_1, Con _2, Con _3) and three from the astaxanthin-supplemented group (As_1, As_2, and As_3). Paired-end sequencing was conducted on the Illumina NovaSeq™ 6000 platform at Lc-bio (Hangzhou, China) following the manufacturer's recommended protocol.

We used Bowtie2 and hisat2 to map reads to the genome of Nile tilapia (https://www.ncbi.nlm.nih.gov/genome/?term=nile+tilapia). Gene expression levels were measured using FPKM (fragments per kilobase of exon model per million mapped reads) to measure the abundance of gene expression. Differentially expressed (DE) genes were analyzed on the basis of fold-change (the mean value of FPKM of the As/the mean value of FPKM of the Con) and *P* value criteria, and then a false discovery rate (FDR) correction was applied to adjust the *P* value. The thresholds for a significant difference in gene transcript levels were |log_2_foldchange| ≥ 1 and *P* < 0.05. The gene ontology (GO; http://www.geneontology.org) and Kyoto Encyclopedia of Genes and Genomes (KEGG) pathway (http://www.genome.jp/kegg/pathway. html) databases were used to assign terms and pathways to the genes to investigate their potential biological functions. On the basis of the sequencing results, several DE genes in important pathways were selected for qRT-PCR verification. For specific procedures, refer to section 2.7 below.

### 2.7. Quantitative Gene Expression Analysis

On the basis of the mRNA sequence of related genes at the NCBI, we designed primers to amplify apoptosis-related genes (B-cell lymphoma 2: *bcl-2*; BCL2 associated X: *bax*, *caspase-3*) and hormone receptor genes (Estrogen receptor: *er*, follicle-stimulating hormone receptor: *fshr*; luteinizing hormone receptor: *lhr*) (Supplementary Table 1). Total RNA was extracted from 50 mg gonad tissue (16 samples in each group) using 1 mL TRIzol (Invitrogen, Carlsbad, CA, USA). The purity and concentration of the extracted RNA were confirmed (OD 260/OD 280: 1.8–2.0; RNA concentration: 55–80 *μ*g/mL) before reverse transcription to produce cDNA using a Prime Script™ II 1st strand cDNA synthesis Kit (TaKaRa, Dalian, China). Gene transcript levels in gonadal tissue were determined by quantitative PCR using the FastStart Universal SYBR Green Master (ROX) Mix (TaKaRa). The Ct value of the target gene was calculated as the mean value of three replicates. *β*-Actin was used as the internal reference. The difference between the Ct value of the target gene and that of the internal reference gene (∆∆Ct) was used to calculate the 2^-∆∆Ct^ value as the relative gene transcript level in each sample. The PCR reaction conditions were as follows: 95°C, 5 min; 95°C, 15 s; and 60°C, 60 s, 40 cycles.

### 2.8. Statistical Analysis

Data were analyzed using SPSS version 25 (SPSS, Chicago, IL, USA). All data were first subjected to Shapiro-Wilk's and Levene's tests to analyze data normality and variance homogeneity, followed by one-way analysis of variance (ANOVA). Significant differences (*P* < 0.05) among groups were further compared using Duncan's multiple range tests. All results are expressed as mean ± SD.

## 3. Results

### 3.1. Effects of Dietary Astaxanthin Levels on Growth Performance of Nile Tilapia

Compared with the control group, the groups fed with 100 mg/kg and 150 mg/kg astaxanthin showed significantly increased WG and SGR (*P* < 0.05) (Table 2). The group fed with 150 mg/kg astaxanthin had the highest FBW, WG, and SGR. Compared with the groups fed with 50 mg/kg, 100 mg/kg, and 150 mg/kg astaxanthin, the group fed with 200 mg/kg astaxanthin had lower FBW, SGR, and WG. However, there was no significant difference in FBW, SGR, and WG between the group fed with 200 mg/kg astaxanthin and the control group (*P* > 0.05). The HSI and VSI of each experimental group increased first and then decreased during the experimental period. The HSI and VSI were significantly higher in the group fed with 150 mg/kg astaxanthin than in the control group and the group fed with 200 mg/kg astaxanthin (*P* < 0.05). There were no significant differences in HSI and VSI among the control and the 50 mg/kg and 200 mg/kg astaxanthin groups. The FCR was significantly lower in the group with 100 mg/kg astaxanthin than in the control group and the group fed with 200 mg/kg astaxanthin (*P* < 0.05).

### 3.2. Effects of Dietary Astaxanthin Levels on Ovarian Development of Nile Tilapia

**Figure 1:**
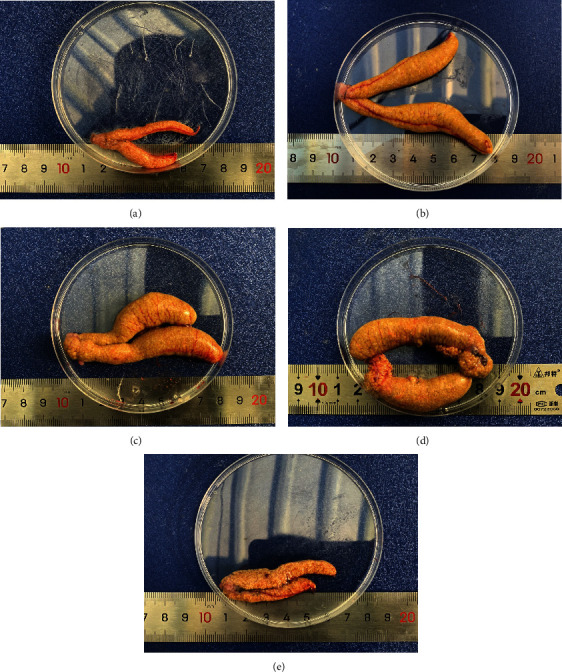
Gonad tissues of Nile tilapia fed with diets containing astaxanthin at different levels for 60 days. Representative images of gonad tissue from female fish fed with diets containing different levels of astaxanthin. (a)–(e) correspond to experimental groups fed with diets containing 0, 50 mg/kg, 100 mg/kg, 150 mg/kg, and 200 mg/kg astaxanthin, respectively.

As shown in Figures 1(c) and 1(d), the ovarian tissues of Nile tilapia in the groups fed with 100 mg/kg and 150 mg/kg astaxanthin were full, and the eggs were yellow-gray, uniform, clear, and countable (Figure 1). The ovarian tissue of the control group was poorly developed, and the eggs were significantly smaller (Figure 1(a)). However, the ovarian tissue of tilapia in the group fed with 200 mg/kg astaxanthin was significantly atrophied and contained more white eggs (Figure 1(e)). The GSI increased with increasing astaxanthin supplementation levels from 0 to 150 mg/kg. However, the GSI in the 200 mg/kg astaxanthin group was significantly lower than those in the 100 mg/kg and 150 mg/kg astaxanthin groups (Table 3, *P* < 0.05), but not significantly different from that in the control group and 50 mg/kg astaxanthin group (*P* > 0.05). The *F*_*R*_ of each experimental group first increased and then decreased with increasing astaxanthin supplementation. The *F*_*R*_ was significantly higher in the group fed with 150 mg/kg astaxanthin than in the control group and the group fed with 200 mg/kg astaxanthin (*P* < 0.05). The egg diameter was significantly higher in the groups fed with 100 mg/kg and 150 mg/kg astaxanthin than in the other groups (*P* < 0.05). There was no significant difference in egg diameter among the control and the 50 mg/kg and 200 mg/kg astaxanthin groups.

**Figure 2:**
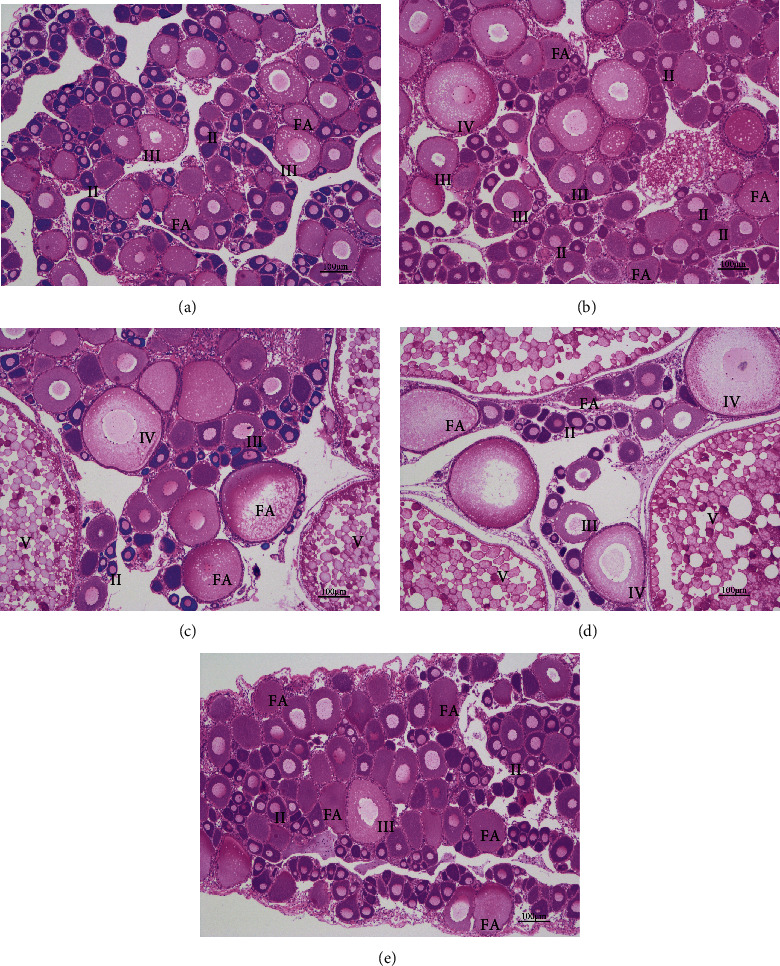
Oocyte development in Nile tilapia fed with diets containing astaxanthin at different levels for 60 days. Representative images of hematoxylin/eosin-stained gonad tissue from female fish fed with diets containing astaxanthin (magnification ×200, scale bar: 100 *μ*m). (a)–(e) correspond to groups fed with diets containing astaxanthin at 0, 50 mg/kg, 100 mg/kg, 150 mg/kg, and 200 mg/kg, respectively. II, III, IV, and V represent oocytes at stages II, III, IV, and V, respectively; FA: follicular atresia.

We observed oocyte development in the fish fed with different levels of astaxanthin (Figure 2). In the control group, most of the oocytes were at stage II and III, and atretic follicles were present (Figure 2(a)). In the group fed with 50 mg/kg astaxanthin, stage IV oocytes were present in the ovary, as well as stages II and III oocytes and atretic follicles (Figure 2(b)). The oocytes of fish in the 100 mg/kg and 150 mg/kg astaxanthin groups had developed to stage V, and there were a few stage II and III oocytes (Figures 2(c) and 2(d)). However, in the 200 mg/kg astaxanthin group, most oocytes were at stages II and III, and many atretic follicles were present (Figure 2(e)). There were significantly more atretic follicles in the 200 mg/kg astaxanthin group than in the other experimental groups (Table 3).

### 3.3. Effects of Dietary Astaxanthin Levels on Antioxidant Capacity of Nile Tilapia

**Figure 3:**
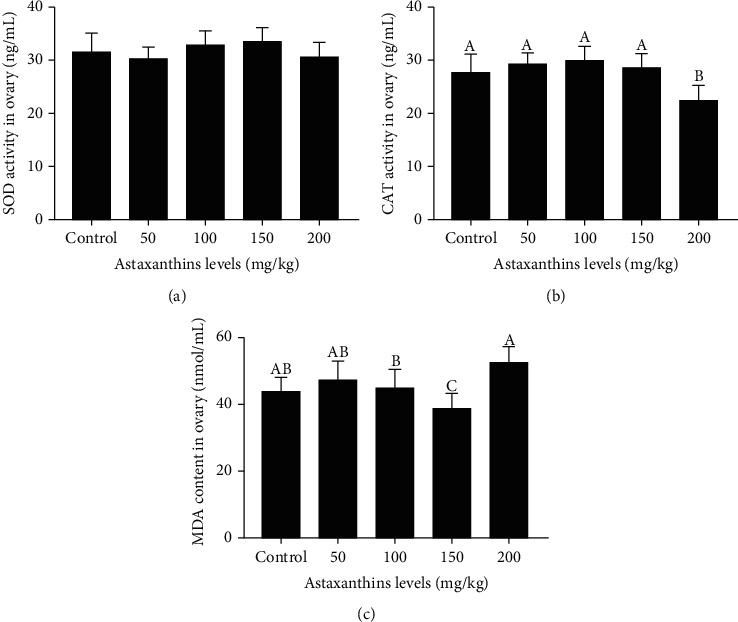
Antioxidant capacity of gonad tissue of Nile tilapia fed with diets containing astaxanthin at different levels for 60 days. Sixteen samples from each group were analyzed in these experiments. Different lowercase letters indicate significant differences among different groups (Duncan's multiple range test; *P* < 0.05).

Different levels of astaxanthin in the feed did not affect SOD activity in Nile tilapia females (Figure 3, *P* > 0.05). The MDA content tended to decrease with increasing levels of astaxanthin supplementation from 0 to 150 mg/kg but was significantly higher in the group fed with 200 mg/kg astaxanthin than in the groups fed with 100 mg/kg and 150 mg/kg astaxanthin (*P* < 0.05). The CAT activity was not significantly different among the control and the 50 mg/kg, 100 mg/kg, and 150 mg/kg astaxanthin groups. However, it was significantly lower in the 200 mg/kg astaxanthin group than in the other groups (*P* < 0.05).

### 3.4. Effects of Dietary Astaxanthin Levels on Serum Hormone Levels of Nile Tilapia

**Figure 4:**
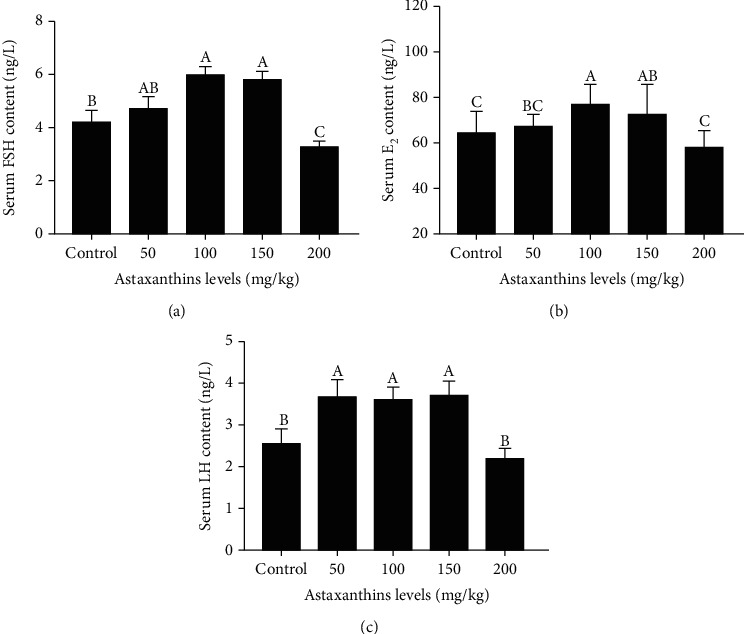
Serum hormone levels in Nile tilapia fed with diets containing astaxanthin at different levels for 60 days. Sixteen samples from each group were analyzed. Different lowercase letters indicate significant differences among different groups (Duncan's multiple range test; *P* < 0.05).

Serum FSH, E_2_, and LH levels were significantly affected by dietary astaxanthin levels (Figure 4, *P* < 0.05). The serum levels of FSH, E_2_, and LH were higher in the groups fed with 100 mg/kg and 150 mg/kg astaxanthin than in the other groups. The serum FSH, E_2_, and LH levels were significantly lower in the 200 mg/kg astaxanthin group than in the 100 mg/kg and 150 mg/kg astaxanthin groups (*P* < 0.05).

### 3.5. Effects of Dietary Astaxanthin Levels on Gene Expression and Apoptosis in Nile Tilapia

**Figure 5:**
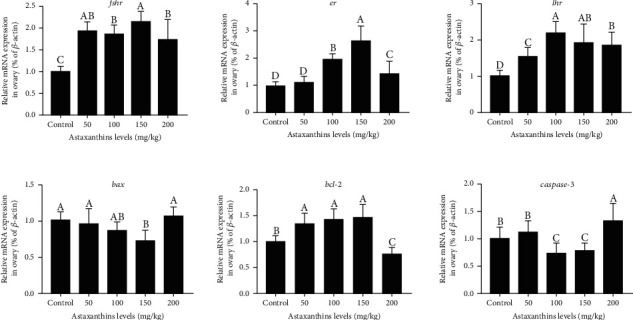
Transcript levels of genes related to hormone receptors and apoptosis in Nile tilapia fed with diets containing astaxanthin at different levels for 60 days. Sixteen samples from each group were analyzed. Different lowercase letters indicate significant differences among different groups (Duncan's multiple range test; *P* < 0.05).

**Figure 6:**
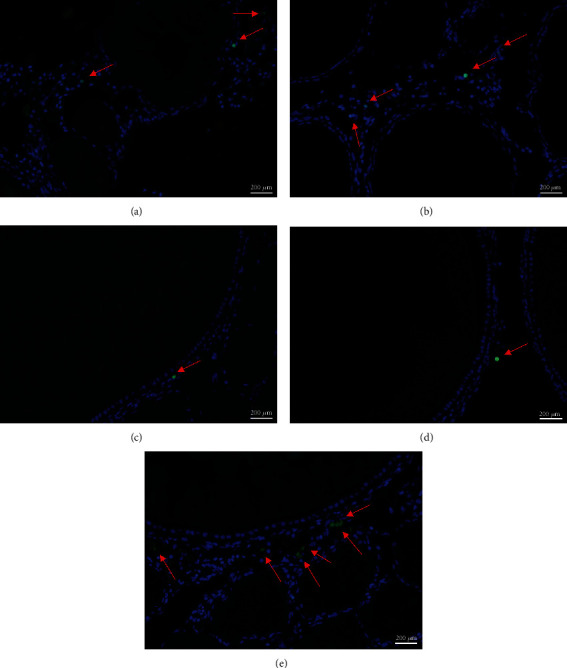
Granulosa cell apoptosis in Nile tilapia fed with diets containing astaxanthin at different levels for 60 days. Representative images of TUNEL-stained gonad tissue from female fish fed with diets containing astaxanthin (magnification ×100, scale bar: 200 *μ*m). (a)–(e) correspond to experimental groups fed with diets containing astaxanthin at 0, 50 mg/kg, 100 mg/kg, 150 mg/kg, and 200 mg/kg, respectively. Nuclei of nonapoptotic cells stained with DAPI fluoresce bright blue under a UV filter; nuclei of apoptotic cells labeled with FITC are green.

The transcript levels of *fshr* and *er* were significantly higher in the groups fed with 100 mg/kg and 150 mg/kg astaxanthin than in the control group and the group fed with 200 mg/kg astaxanthin (Figures 5(a) and 5(b), *P* < 0.05). The transcript levels of *lhr* were significantly higher in the groups fed with 100 mg/kg, 150 mg/kg, and 200 mg/kg astaxanthin than in the control group and the group fed with 50 mg/kg astaxanthin (Figure 5(c)). However, there was no significant difference in *lhr* transcript levels among the groups fed with 100 mg/kg, 150 mg/kg, and 200 mg/kg astaxanthin (*P* > 0.05). The levels of *bax* and *caspase-3* were significantly higher in the control group and the group fed with 200 mg/kg astaxanthin than in the group fed with 150 mg/kg astaxanthin (Figures 5(d) and 5(f)). The transcript level of *bcl-2* was significantly lower in the group fed with 200 mg/kg astaxanthin than in the groups fed with 50 mg/kg, 100 mg/kg, and 150 mg/kg astaxanthin (Figure 5(e)). In the TUNEL analyses, there were more green positive apoptotic cells around the follicles in the control group and the group fed with 200 mg/kg astaxanthin than in the other groups (Table 4; Figures 6(a) and 6(e), *P* < 0.05). However, there were significantly more granulosa cell layers and fewer apoptotic cells in the groups fed with 100 mg/kg and 150 mg/kg astaxanthin than in the control group and the group fed with 50 mg/kg astaxanthin (Figures 6(c) and 6(d), *P* < 0.05).

### 3.6. Transcriptome Analysis to Reveal Molecular Mechanism by which Dietary Astaxanthin Regulates Ovarian Development in Nile Tilapia

**Figure 7:**
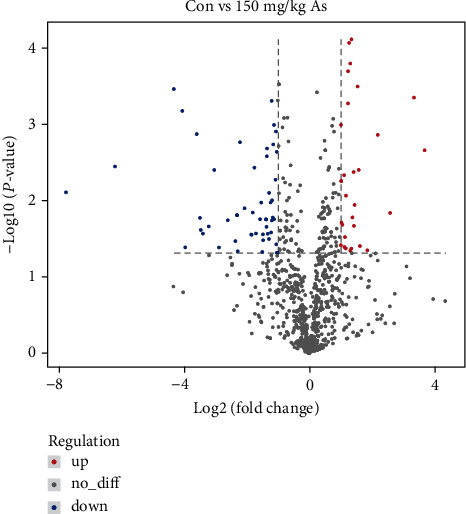
Number of differentially expressed genes in Nile tilapia ovarian tissue between control group and group fed with a diet containing 150 mg/kg astaxanthin. Note: RNA from four samples was mixed to construct each sequencing library. Six sequencing libraries were constructed, three from control groups (Con) and three from 150 mg/kg astaxanthin groups (150 mg/kg As).

**Figure 8:**
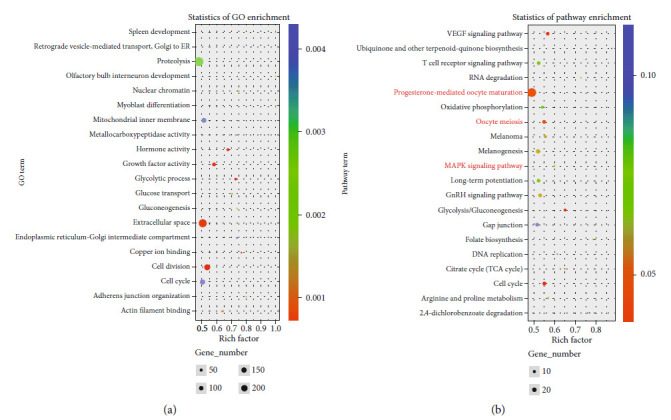
Gene ontology classification and pathway enrichment analysis of differentially expressed genes in gonad tissue between control group (Con) and 150 mg/kg astaxanthin group (150 mg/kg As). (a) Gene ontology classification of putative functions of differentially expressed genes in Con vs. 150 mg/kg As. (b) KEGG enrichment subclass and signal pathways of differentially expressed genes in Con vs. 150 mg/kg As.

The control group and the optimal astaxanthin-supplemented group (150 mg/kg As) were selected for RNA-seq analysis. We detected 462 DE mRNAs (*P* ≤ 0.05,|Log2fold change| ≥ 1) between the two groups, of which 202 were upregulated and 260 were downregulated (Figure 7). The GO term enrichment analysis showed that the DE genes (Con *vs*. 150 mg/kg As) detected from the RNA-seq data were mainly related to the proteolysis, hormone activity, growth factor activity, extracellular space, cell division, and cell cycle (Figure 8(a)). We conducted KEGG analyses of the DE mRNAs in the control group *vs.* 150 mg/kg As. These analyses indicated that astaxanthin regulates ovarian development *via* its effects on cell fate control (MAPK signaling, cell cycle), oocyte development and maturation (gap junction, oocyte meiosis, and progesterone-mediated oocyte maturation), metabolism (oxidative phosphorylation, glycolysis/gluconeogenesis, arginine, and proline metabolism), and hormone regulation (GnRH signaling pathway) (Figure 8(b)). Among these pathways, the MAPK signaling pathways, which affects apoptosis, as well as oocyte meiosis and progesterone-mediated oocyte maturation were identified as being primarily involved in the astaxanthin-induced regulation of follicular development in tilapia. These signaling pathways were the focus of further analyses.

**Figure 9:**
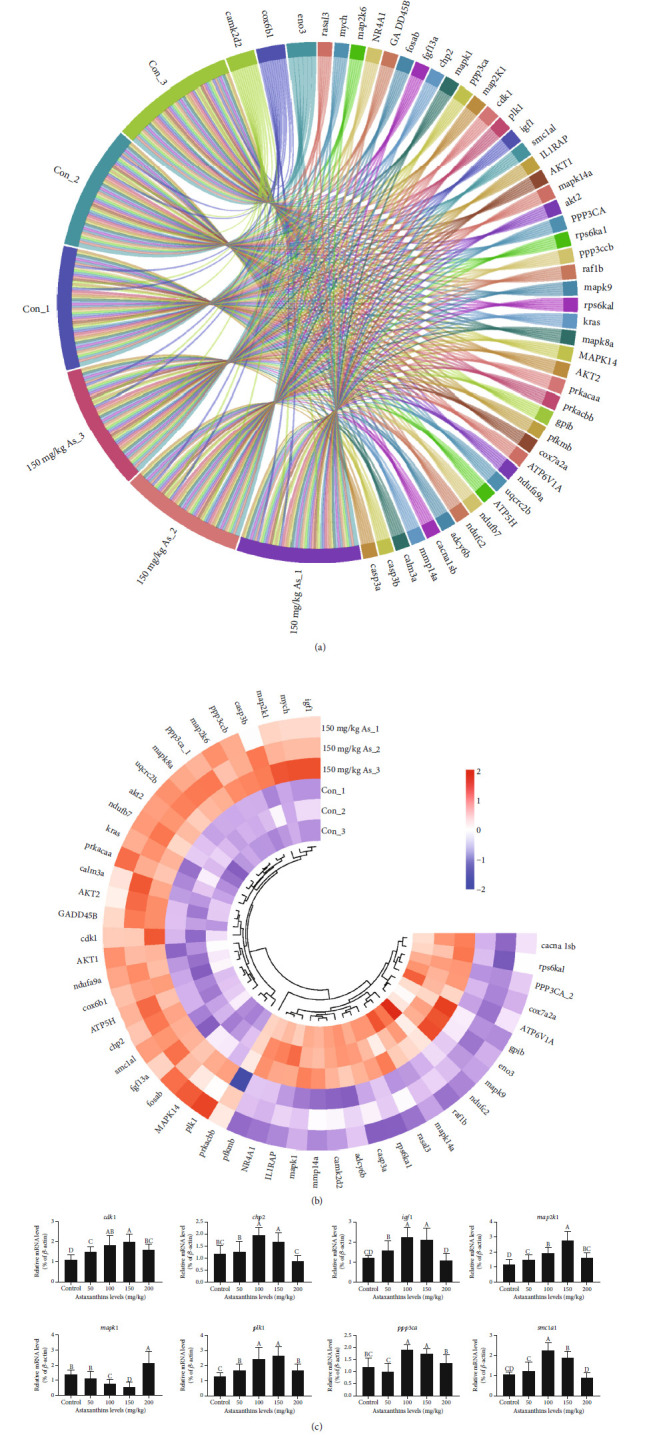
Representative differentially expressed genes involved in regulation of follicular development (control group *vs.* 150 mg/kg astaxanthin group). Note: (a) Circular representation of 51 differentially expressed genes associated with regulation of follicular development in control group (Con) *vs.* 150 mg/kg astaxanthin group (150 mg/kg As). (b) Transcript levels of 51 differentially expressed genes associated with regulation of follicular development in Con *vs.* 150 mg/kg As as determined from transcriptome data. Red and blue indicate upregulated and downregulated genes, respectively. (c) Transcript levels of key genes in oocyte meiosis, progesterone-mediated oocyte maturation, and MAPK signaling pathways by qRT-PCT. Different lowercase letters indicate significant differences among different groups (Duncan's multiple range test; *P* < 0.05).

Notably, we identified 51 significant DE genes involved in follicular development, metabolic regulation, apoptosis, and adaptive immunity in the fish fed with a diet containing 150 mg/kg astaxanthin compared with the control group (Figure 9(a), Supplementary Table 2). The hierarchical clustering heatmap divided these DE genes into two major clusters (Con *vs.* 150 mg/kg As) (Figure 9(b)). Quantitative analyses of eight DE genes involved in these pathways revealed that *mapk1* (mitogen-activated protein kinase 1) was significantly downregulated in ovarian tissue in the 100 mg/kg and 150 mg/kg astaxanthin groups compared with the control group; and that *chp2* (calcineurin B homologous protein 2), *ppp3ca* (protein phosphatase 3 catalytic A), *map2k1* (mitogen-activated protein kinase kinase 1), *cdk1* (cyclin-dependent kinase 1), *plk1* (serine/threonine-protein kinase), *igf1* (insulin-like growth factor I), and *smc1a1* (structural maintenance of chromosomes protein 1A) were significantly upregulated in the 100 mg/kg and 150 mg/kg astaxanthin groups (Figure 9(c)). Among them, *chp2*, *mapk1*, *ppp3ca*, and *map2k1* encode components of the MAPK signaling pathway involved in oocyte meiosis; *cdk1*, *plk1*, and *igf1* encode members of multiple follicular development regulation pathways (oocyte meiosis and progesterone-mediated oocyte maturation); and *smc1a1* is involved in oocyte meiosis. Compared with the 100 mg/kg and 150 mg/kg astaxanthin groups, the 200 mg/kg astaxanthin group showed significant upregulation of *mapk1*. In contrast, the transcript levels of *ppp3ca*, *chp2*, *map2k1*, *cdk1*, *plk1*, *igf1*, and *smc1a1* were all significantly lower in the 200 mg/kg astaxanthin group than in the 150 mg/kg astaxanthin group (*P* < 0.05).

## 4. Discussion

### 4.1. Dietary Supplementation with 150 mg/kg Astaxanthin Promoted the Growth of Nile Tilapia

Astaxanthin has a growth-promoting effect on aquacultured animals. Previous studies have shown that dietary supplementation with astaxanthin at 200–300 mg/kg can significantly increase the weight gain of discus fish (*Symphysodon haraldi*) [20] and that a diet containing 200 mg/kg astaxanthin can increase the growth performance of golden pompano (*Trachinotus ovatus*) and oscar [2, 21]. In this study, the optimal doses of astaxanthin (150 mg/kg) significantly increased the growth and reduced the FCR of Nile tilapia. However, the highest dose (200 mg/kg) inhibited the growth and feed utilization of the broodstock. Excessive astaxanthin may increase metabolism in the fish body, resulting in additional physical energy demands to excrete excess nutrients from the body [22]. Diets supplemented with 500 mg/kg natural astaxanthin (*Haematococcus pluvialis* extract) [20] or 300–400 mg/kg synthetic astaxanthin were also found to significantly reduce the weight gain of discus fish [23]. The effect of astaxanthin on the growth performance of fish may be related to factors such as the fish species, growth stage, feed composition, and rearing conditions.

### 4.2. Dietary Supplementation with 150 mg/kg Astaxanthin Promoted Follicular Development of Nile Tilapia

Successful gonad development and the hatching rate of fertilized eggs are affected by the culture methods and broodstock feed. The reproductive performance of broodstock can be significantly improved by supplementing the diet with high-quality protein sources, large amounts of unsaturated fatty acids, and vitamin A ([24–26]). The GSI is a reliable indicator of the status of gonad development. Increasing protein levels in the feed or adding nutrients can significantly improve the GSI and fertility of broodstock [26, 27]. In the present study, dietary supplementation with astaxanthin at 150 mg/kg not only promoted the growth and feed utilization of Nile tilapia, but also stimulated ovarian development. Also, the HSI and GSI were positively correlated during gonad development. The oocytes in the 100 mg/kg and 150 mg/kg astaxanthin groups were mainly in the middle and late stages of vitellogenesis (stages IV and V). The main process at this stage is the synthesis of exogenous yolk, and the exogenous substances are mainly from the liver [24]. A higher HSI may contribute to the continuous transport of nutrients to the ovary, increase GSI, and promote follicular development [28].

Studies have shown that the appropriate amount of astaxanthin in the feed can increase the spawning rate of broodstock and the survival of larvae after fertilization [14]. The egg diameter reflects the quality of fish eggs to a certain extent, and the quality affects the early development and survival of fertilized eggs. Therefore, egg diameter is commonly used to evaluate the quality of fish eggs [29]. In this study, the diets with 100 mg/kg and 150 mg/kg astaxanthin effectively improved the fecundity of Nile tilapia broodstock and increased the egg diameter. In another study, freshwater crayfish (*Astacus leptodactylus*) were fed with diets supplemented with vitamins E, C, and A, astaxanthin, and *β*-carotene, and those fed with diets containing vitamin E and astaxanthin produced the most and the largest eggs [12]. In a study on female grass shrimp (*Penaeus monodon*), a diet containing 50 mg/kg astaxanthin increased absolute fecundity and the total spawning numbers [25]. Astaxanthin is a carotenoid and is a precursor of vitamin A. Therefore, an appropriate amount of astaxanthin in the diet (100–150 mg/kg) may stimulate the growth and development of tilapia by increasing vitamin A synthesis. In this study, however, 200 mg/kg astaxanthin in the feed resulted in lower HSI, GSI, *F*_*R*_, and egg size of the female fish, compared with those in the 150 mg/kg astaxanthin group, and this significantly hindered development. Furuita et al. [24] also found that dietary supplementation with excess vitamin A significantly inhibited the growth, HSI, and GSI of Japanese flounder (*Paralichthys olivaceus*), but did not affect egg quality. How excessive astaxanthin suppresses ovarian development will be the focus of further discussion.

### 4.3. Dietary Supplementation with 150 mg/kg Astaxanthin Reduced Oxidative Stress in Nile Tilapia and Alleviated Apoptosis

Astaxanthin is able to eliminate superoxide anion free radicals because of its unique molecular structure. It is a stronger and more effective antioxidant than *β*-carotenoids and vitamin E [12]. Cui et al. [30] found that rainbow trout (*Oncorhynchus mykiss*) fed with appropriate amounts of astaxanthin and canthaxanthin showed increased hepatic total antioxidant capacity. Wang et al. [22] also found that the activity of antioxidant enzymes in serum was significantly increased after koi carp were fed with a diet supplemented with astaxanthin. In this study, dietary supplementation with 150 mg/kg astaxanthin significantly increased CAT activity in the ovarian tissue of female Nile tilapia and reduced the MDA content. The accumulation of MDA, which is the reaction product of lipid peroxidation, is an indicator of oxidative damage. The number of conjugated bonds in the structure of astaxanthin is related to its strong antioxidant function, which allows it to effectively remove oxygen free radicals from ovarian tissue and reduce body damage [31]. Under normal conditions, a certain amount of reactive oxygen species is produced in the follicles, and these molecules play an important role in follicle development and ovulation [32]. Dietary supplementation with astaxanthin improves antioxidant capacity and reduces oxidative damage in the ovary during gonad development in tilapia. Dietary supplementation with vitamin A can also increase the antioxidant capacity of the ovaries and increase the transport of unsaturated fatty acids [33]. However, excessive astaxanthin may lead to increased metabolism in fish, and excess free radical production can cause oxidative damage to biological macromolecules and induce cell apoptosis. The results of TUNEL and HE analyses show that the fish fed with a diet containing 200 mg/kg astaxanthin displayed significantly increased granulosa cell apoptosis and follicular atresia. This may have been caused by oxidative stress resulting from excess astaxanthin.

### 4.4. Dietary Supplementation with 150 mg/kg Astaxanthin Increased Serum Hormone Contents and Transcript Levels of Their Receptor Genes in Nile Tilapia and Alleviated Apoptosis

In this study, supplementation of the feed with 50–150 mg/kg astaxanthin significantly increased the levels of sex steroid hormones in tilapia broodstock. Thus, the appropriate amount of astaxanthin may accelerate the synthesis of sex steroid hormones, thereby promoting gonad maturation. The most important and active form of estrogen is E_2_, which initiates ovarian differentiation and regulates oocyte development and maturation [34]. In studies on African catfish (*Clarias gariepinus*) [35], Channel catfish (*Ictalurus punctatus*) [36], and Pacific cod (*Gadus macrocephalus*) [37], the level of E_2_ was found to be positively correlated with the accumulation of egg yolk in oocytes. Babin et al. [38] found that in female broodstock, the plasma E_2_ content increased during yolk production and then decreased during maturation. Few studies have explored the ability of astaxanthin to promote the synthesis of sex steroid hormones. However, astaxanthin, as the precursor of vitamin A, may promote the synthesis of sterol hormones and follicular development in female fish by increasing vitamin A synthesis. The lack of vitamin A in broodstock feeds can cause gonad development disorders [28]. Appropriate vitamin A intake was shown to increase the fecundity of bighead carp (*Aristichthys nobilis*) [39] and Japanese flounder [40] and to satisfy the energy and nutritional requirements for gonadal development. However, excess astaxanthin can inhibit serum E_2_ levels and follicular development. Similar results were obtained in studies on rainbow trout and tongue sole (*Cynoglossus semilaevis*) broodstock fed with diets containing high levels of vitamin A [28, 41].

Both LH and FSH are synthesized in the pituitary gland and regulate follicle production and subsequent sex hormone production in female fish (e.g. E_2_). The levels of FSH and LH in juvenile brook trout (*Salvelinus fontinalis*) [42] and yellow catfish (*Pelteobagrus fulvidraco*) [43] were found to steadily increase during gonad development and to peak at maturity. In the present study, the HE staining analyses revealed abundant IV and V oocytes in the ovaries of the 100 mg/kg and 150 mg/kg astaxanthin groups and a large number of mature follicles. This was related to the higher serum LH and FSH levels. Also, the increased transcript levels of *fshr* and *lhr* suggest that FSH and LH mediate ovarian development and follicular maturation *via* their corresponding receptors [44], ultimately reducing follicular atresia. The physiological functions of estrogen are indirectly regulated by ER, which is a member of the nuclear receptor family of steroid hormones. Previous studies have shown that changes in the contents of ERa and E_2_ are related and that E_2_ can increase the transcript levels of *er* genes [45]. An appropriate amount of astaxanthin may increase the binding rate of ER and E_2_ and promote ER expression [46]. Enriched FSH, LH, and E_2_ can promote follicular development by alleviating the apoptosis of granulosa cells. In addition, dietary supplementation with 100 mg/kg and 150 mg/kg astaxanthin inhibited the expression of apoptosis genes (*caspase-3* and *bax*) and promoted the expression of the antiapoptosis gene *bcl-2*. Such changes in gene expression have been shown to reduce apoptosis of granulosa cells [47]. However, we found that excess astaxanthin not only inhibited the synthesis and secretion of FSH, LH, and E_2_, but also reduced the activity of hormone receptors. This may have affected the sensitivity of oocytes to hormones, thereby accelerating granular cell apoptosis and follicular atresia.

### 4.5. Dietary Supplementation with 150 mg/kg Astaxanthin Reduced Follicular Atresia by Regulating MAPKs Signal

The maturation or atresia of the follicle depends on the dynamic balance of signaling molecules at different stages, and the granulosa cell is an important intermediary *via* which these signal molecules affect follicle development [16]. Our analyses of oocyte morphology, serum hormone contents, and oxidative stress levels revealed that the 150 mg/kg astaxanthin group showed reduced ovarian oxidative stress, increased hormone secretion and expression of receptor genes, and alleviation of granular cell apoptosis, leading to better follicular development. Further analyses of the transcriptome revealed details of the molecular mechanism by which dietary astaxanthin at 150 mg/kg promoted follicular development.

MAPKs signal transduction pathways are involved in the regulation of cell growth, reproduction, division, and death and various biochemical reactions in the cell [48]. In the present study, analyses of the transcript levels of *mapk1* (encoding MAP kinase) and its down-stream targets (*chp2*, *map2k1*, and *ppp3ca*) revealed that MAPK signaling was involved in reducing granulosa cell apoptosis in the 150 mg/kg astaxanthin group. *Chp2* participates in the regulation of cell proliferation, and reducing the expression of its encoding gene or inhibiting its activity can accelerate cell death. Inhibition of chp2 in breast cancer cells can delay the G1-S cell cycle transition [49]. Both ppp3ca and chp2 are calcineurins [50]. The upregulation of *ppp3ca* and *chp2* in the 150 mg/kg astaxanthin group may affect the transmission of intracellular calcium signals, leading to the alleviation of cell apoptosis and activation of oocyte meiosis. As a negative regulator of MAPK signaling, mapk1 is closely related to the control of cell fate because it triggers apoptosis [51]. In another study, the upregulation of map2k1was found to alleviate apoptosis of LPS-induced WI-38 cells and reduce cell damage and inflammation [52]. Therefore, the downregulation of *mapk1* and upregulation of *map2k1* in the 150 mg/kg astaxanthin group may have promoted the proliferation of granulosa cells and follicle development. However, excessive astaxanthin may increase the metabolic burden in the fish body, increase oxidative stress, stimulate the MAPK signaling pathway, aggravate cell damage and apoptosis, and induce follicular atresia.

Oocyte meiosis and progesterone-mediated oocyte maturation are important signaling pathways that regulate follicular development. If the expression of *cdk1* is reduced, the cell cycle is significantly blocked at the G2/M phase. Disorders of the cell cycle and long-term cell cycle arrest are important inducers of cell apoptosis [53]. Plk1 is a regulated protein kinase involved in oocyte meiosis and also in multiple steps during mitosis such as the cell G2/M phase transition and chromosome segregation [54]. Smc1a1 is related to cell proliferation, signal transmission, and the maintenance of chromosome stability [55]. Female fish consuming an appropriate amount of astaxanthin were found to show increased transcript levels of *cdk1*, *plk1*, and *smc1a1*; enhanced differentiation and proliferation of granulosa cells; increased transmission of various cytokines, hormones, and growth factor signals to oocytes; and improved follicular development [56]. The increased amount of atretic follicles in the 200 mg/kg astaxanthin group may be related to the abnormal expression of *chp2*, *cdk1*, and *plk1* leading to disordered granulosa cell proliferation and increased apoptosis.

Granulosa cell apoptosis and follicular atresia are regulated by insulin-like growth factors (IGFs) [57]. As a cofactor of gonadotropin, igf1, together with FSH, stimulates granulosa cells/luteal cells to produce estradiol and progesterone, and promotes the proliferation of granulosa cells and follicular membrane cells [58]. In the present study, the upregulation of *igf1* in the 100 mg/kg and 150 mg/kg astaxanthin groups and the higher serum FSH, LH, and E_2_ contents promoted oocyte meiosis and progesterone-mediated oocyte maturation signaling pathways, which enhanced follicular development.

## 5. Conclusion

In this study, Nile tilapia female fed with a diet containing 150 mg/kg astaxanthin showed improved follicular development as a result of increased serum estrogen content and reduced oxidative stress. The results of mRNA sequencing and TUNEL analyses revealed that the diet containing 150 mg/kg astaxanthin resulted in alleviation of cell apoptosis and increased granulosa cell proliferation and follicle maturation. In contrast, a high dose of astaxanthin (200 mg/kg) promoted MAPK signaling, which triggered granular cell apoptosis in the ovary and accelerated follicular atresia. The results of our study can be applied to increase the spawning efficiency of Nile tilapia broodstock and to prevent or alleviate ovarian stress.

## Figures and Tables

**Table 1:** The formulation and chemical composition of the experimental diets.

Dietary	Astaxanthin levels (mg/kg)				
Ingredients	Control	50	100	150	200
Fish meal	100	100	100	100	100
Wheat middling	106	106	106	106	106
Corn starch	168	168	168	168	168
Soybean oil percentage sign removed	50	50	50	50	50
Soybean meal	160	160	160	160	160
Cottonseed meal	160	160	160	160	160
Rapeseed meal	160	160	160	160	160
Compound vitamin	5	5	5	5	5
Compound mineral salt	5	5	5	5	5
Choline chloride	5	5	5	5	5
Vitamin C phosphate sodium	2	2	2	2	2
Calcium dihydrogen phosphate	15	15	15	15	15
Microcrystalline cellulose	64	63.5	63	62.5	62
*Haematococcus pluvialis*	0	0.5	1	1.5	2
Total	1000	1000	1000	1000	1000
*Proximate composition*					
Crude protein (%)	29.4	29.2	29.6	29.4	29.6
Crude lipid (%)	6.8	6.8	6.7	6.7	6.7
Astaxanthin (mg/kg)	0	43.93	89.29	134.46	179.33

Note: Fish meal was got from the Copeinca (Lima, Peru), soya bean meal was obtained from Tongwei Feed Corporation Ltd. (Wuxi, China), rapeseed meal was obtained from Tongwei Feed Corporation Ltd. (Wuxi, China), vitamins and minerals mix were supplied by Guangzhou Chengyi Aquatic Technology Ltd. (Guangzhou, China).

**Table 2:** Effects of dietary astaxanthin levels on the growth performance of Nile tilapia females (*Oreochromis niloticus*).

	Astaxanthin levels (mg/kg)				
	Control	50	100	150	200
FBW (g/fish)	423.79 ± 18.96^b^	459.15 ± 16.36^ab^	463.03 ± 18.77^a^	468.1 ± 14.44^a^	436.72 ± 14.98^ab^
SGR (%/day)	1.18 ± 0.32^b^	1.32 ± 0.18^a^	1.34 ± 0.22^a^	1.37 ± 0.14^a^	1.23 ± 0.17^ab^
WG (%)	86.48 ± 7.35^b^	102.03 ± 8.12^a^	103.78 ± 6.66^a^	105.97 ± 6.28^a^	92.16 ± 6.52^b^
HSI (%)	1.56 ± 0.4^c^	1.79 ± 0.44^b^	1.82 ± 0.34^ab^	1.96 ± 0.65^a^	1.76 ± 0.43^b^
VSI (%)	7.56 ± 1.26^c^	8.36 ± 1.75^b^	8.61 ± 1.49^ab^	9.06 ± 1.99^a^	7.97 ± 1.36^bc^
FCR	1.30 ± 0.13^a^	1.28 ± 0.14^ab^	1.18 ± 0.18^c^	1.25 ± 0.12^bc^	1.33 ± 0.13^a^

Note: Data are Means ± SD (*n* = 16). Values in the same row with different superscripts are significantly different by Duncan's test (*P* < 0.05).

**Table 3:** Effects of dietary astaxanthin levels on the gonadal development of Nile tilapia females (*Oreochromis niloticus*).

	Astaxanthin level (mg/kg)				
	Control	50	100	150	200
GSI (%)	1.86 ± 0.59^c^	2.46 ± 0.62^b^	3.02 ± 0.99^a^	3.13 ± 0.7^a^	2.43 ± 0.97^b^
Relative fecundity	16.14 ± 6.32^b^	17.28 ± 6.27^a^	17.08 ± 5.71^a^	17.99 ± 5.18^a^	16.32 ± 5.82^b^
(eggs kg^−1^ body weight)					
Egg diameter (mm)	1.65 ± 0.42^c^	1.87 ± 0.46^b^	2.32 ± 0.32^a^	2.33 ± 0.41^a^	1.72 ± 0.39^bc^
The number of atretic oocytes	43-71	45-72	38-65	44-69	59-92
Mean of atretic oocytes	57^b^	58^b^	51^c^	57^b^	76^a^

Note: Data of GSI and Relative fecundity are Means ± SD (*n* = 16). Values in the same row with different superscripts are significantly different by Duncan's test (*P* < 0.05).

**Table 4:** Effects of dietary astaxanthin levels on the number of apoptotic cells in the ovary of Nile tilapia females (*Oreochromis Niloticus*).

	Astaxanthin level (mg/kg)				
	Control	50	100	150	200
The number of apoptotic cells	42-68	23-49	3-9	4-8	35-56
Mean of apoptotic cells	55^b^	36^b^	6^c^	6^c^	46^a^

Note: Values in the same row with different superscripts are significantly different by Duncan's test (*P* < 0.05).

## Data Availability

All data was submitted in the Supplementary Information files alongside manuscript.

## Abbreviations

FFRC: Freshwater Fisheries Research Center of the Chinese Academy of Fishery Sciences
TUNEL: Terminal dUTP nick-end labeling
HE: Hematoxylin/eosin
DO: Dissolved oxygen
WG: Weight gain
SGR: Specific growth rate
FCR: Feed conversion ratio
HSI: Hepatosomatic index
VSI: Viscerosomatic index
GSI: Gonadosomatic index
*F*_R_: Relative fecundity
MDA: Malondialdehyde
SOD: Superoxide dismutase
CAT: Catalase
FA: Follicular atresia
FSH: Follicle-stimulating hormone
LH: Luteinizing hormone
*fshr*: FSH receptor gene
*er*: Estrogen receptor
*lhr*: Luteinizing hormone receptor
GO: Gene ontology
KEGG: Kyoto Encyclopedia of Genes and Genomes
Con: Control group
As: Astaxanthin-supplemented group
FBW: Final body weight
IBW: Initial body weight
ELISA: Enzyme-linked immunosorbent assay
FITC: Fluorescein isothiocyanate
DAPI: 4,6-diamidino-2-phenylindole
DE genes: Differentially expressed genes
FDR: False discovery rate
*mapk1*: Mitogen-activated protein kinase 1
*chp2*: Calcineurin B homologous protein 2
*ppp3ca*: Protein phosphatase 3 catalytic A
*map2k1*: Mitogen-activated protein kinase kinase 1
*cdk1*: Cyclin-dependent kinase 1
*plk1*: Serine/threonine-protein kinase
*igf1*: Insulin-like growth factor I
*smc1a1*: Structural maintenance of chromosomes protein 1A.

## Supplementary Materials

Supplementary 1 Table S1: the primer sequence of the gene in this experiment.

Supplementary 2 Table S2: differentially expressed mRNA screening of Nile tilapia (*Oreochromis niloticus*) between the 150 mg/kg astaxanthin group and the control group.
