# Supplementary material for: Role of Astaxanthin as a Stimulator of Ovarian Development in Nile Tilapia (Oreochromis niloticus) and Its Potential Regulatory Mechanism: Ameliorating Oxidative Stress and Apoptosis
Source: Aquac Nutr. 2022 Sep 10;2022:1245151. doi: 10.1155/2022/1245151 (PMC10164245; doi:10.1155/2022/1245151)
Supplement: Supplementary 1 — Table S1: the primer sequence of the gene in this experiment. [file 1245151.f1.docx]

Supplementary Table 1 The primer sequence of the gene in this experiment

| Gene abbreviation | Sequence | NCBI reference |
| --- | --- | --- |
| *bax* | F:5’-ATTCGCTCCTACTTCGGCAC-3’ | XM_019357746.2 |
|  | R:5’-GTTCTTCCTGTTGAGCGGGT-3’ |  |
| *caspase-*3 | F:5’- TGAATTTCCGGGCCTGAGTG-3’ | NM_001282894.1 |
|  | R:5’-GACAGACCGTCATCGTGCTT-3’ |  |
| *bcl*-2 | F:5’-GACGATGATGCCAGGGAGAG-3’ | XM_003437902.5 |
|  | R:5’- CTCAGAGTTCACTGGAGCGG-3’ |  |
| *er* | F:5’- GCCTCCTACACAACCAAGGG-3’  R:5’-CGGTGGTTCTGGTTTGGCTA-3’ | NM_001279770.1 |
| *fshr* | F:5’- GTGTAGTGCATCCGGCAGAG-3’  R:5’-TTCTGTTTGGGCCAGTCGTC-3’ | NM_001279588.1 |
| *lhr* | F:5’- ACAAGCTGACAGTACCTCGC-3’  R:5’-CTGACAGCTCCCCACCAAAA-3’ | XM_005474002.4 |
| *mapk1* | F:5’- ATGGCCATTTTTGCTGGCTG-3’  R:5’- AGGAAGCTGCGTGCTGTTAT-3’ | XM_003444474.5 |
| *chp2* | F:5’- TGAGAACGATTCCCTGTGGC-3’  R:5’- TCACCTCAGAATCACGCTGC-3’ | XM_005474884.4 |
| *ppp3ca* | F:5’- TGCAGAATGCAATCAAAGGCT -3’  R:5’- TTGAGAGAGTTGAGGCTGGC -3’ | XM_005468091.4 |
| *map2k1* | F:5’- ACGGGGCTTTTTACAGCGAT-3’  R:5’- CTGTCCGCTCACTCCAAAGT-3’ | XM_003440445.5 |
| *cdk1* | F:5’- GCTGATTGACAACAAGGGCG-3’  R:5’- ATATCGGGGTGACCCTAGCA-3’ | XM_003454997.5 |
| *plk1* | F:5’- CCGCAATTACATGAGCGAGC-3’  R:5’- ATTGATCTGAACCGTGCCGT-3’ | XM_013265752.3 |
| *igf1* | F:5’- ATGTGATGTCTTCAAGAGTGCGA-3’  R:5’- GACGCACAGCAGTAGTGAGA-3’ | NM_001279503.1 |
| *smc1a1* | F:5’- CTACAAGGCTCTGTCACGCA-3’  R:5’- CAGAGCTGCGTCGATCTCAT-3’ | XM_003448166.5 |
| *β*-actin | F:5’- CCACACAGTGCCCATCTACGA-3’  R:5’- CCACGCTCTGTCAGGATCTTCA -3’ | XM_003443127.5 |
