# Supplementary material for: Role of Astaxanthin as a Stimulator of Ovarian Development in Nile Tilapia (Oreochromis niloticus) and Its Potential Regulatory Mechanism: Ameliorating Oxidative Stress and Apoptosis
Source: Aquac Nutr. 2022 Sep 10;2022:1245151. doi: 10.1155/2022/1245151 (PMC10164245; doi:10.1155/2022/1245151)
Supplement: Supplementary file 2 [file 1245151.f2.docx]

gene_id	gene_name	transcr	GO	KEGG	KO_ENTR	EC	Descrip	150 mg/k	150 mg/kg	150 mg/k	Con_1	Con_2	Con_3	fc	log2(fc	pval
MSTRG.115	rasal3	MSTRG.1	GO:0005	04013(MAPK signaling pathway)	K17633	NA		1.04	2.35	1.23	4.55	2.77	4.17	0.47	-1.10	0.01
MSTRG.120	mych	MSTRG.1	GO:0005	04010(MAPK signaling pathway);04012(Erb	K04377	NA		9.17	10.27	14.07	4.69	6.28	4.67	2.01	1.00	0.02
MSTRG.139	map2k6	ENSONIT	GO:0005	04010(MAPK signaling pathway);04620(Tol	K04433	EC:2.7.12.2		36.50	26.15	37.16	6.98	6.77	5.78	4.89	2.29	0.00
MSTRG.162	NR4A1 (1 of	ENSONIT	GO:0005	04010(MAPK signaling pathway)	K04465	NA		4.07	6.28	7.16	25.60	32.13	22.74	0.24	-2.04	0.01
MSTRG.166	GADD45B (1	ENSONIT	GO:0000	04010(MAPK signaling pathway);04110(Cel	K04402	NA		28.13	39.55	37.47	16.34	14.07	13.61	2.21	1.14	0.01
MSTRG.203	fosab	ENSONIT	GO:0005	04010(MAPK signaling pathway);04620(Tol	K04379	NA		53.86	32.60	30.36	14.00	5.38	5.90	4.74	2.25	0.03
MSTRG.850	fgf13a	MSTRG.8	GO:0008	04010(MAPK signaling pathway);04810(Reg	K04358	NA		3.55	5.95	4.24	0.46	1.14	1.96	2.33	1.22	0.02
MSTRG.127	chp2	ENSONIT	GO:0005	04010(MAPK signaling pathway);04020(Cal	K06268	NA		19.87	34.08	25.38	4.58	2.87	5.56	4.55	2.19	0.00
MSTRG.775	mapk1	ENSONIT	GO:0005	04010(MAPK signaling pathway);04012(Erb	K04371	EC:2.7.11.24		42.19	48.15	33.52	145.28	114.45	119.78	0.35	-1.50	0.03
MSTRG.102	ppp3ca	ENSONIT	GO:0005	04010(MAPK signaling pathway);04020(Cal	K04348	EC:3.1.3.16		12.85	17.82	13.13	3.06	3.28	2.66	3.71	1.89	0.00
MSTRG.347	map2k1	ENSONIT	GO:0005	04010(MAPK signaling pathway);04012(Erb	K04368	EC:2.7.12.2		3.99	5.66	12.00	0.15	0.18	0.22	6.53	2.71	0.01
MSTRG.196	cdk1	ENSONIT	GO:0005	04110(Cell cycle);04114(Oocyte meiosis)	K02087	EC:2.7.11.22;E		6.75	6.84	16.51	1.10	0.20	2.10	5.07	2.34	0.04
MSTRG.231	plk1	ENSONIT	GO:0005	04110(Cell cycle);04114(Oocyte meiosis)	K06631	EC:2.7.11.21		71.63	27.31	48.58	16.30	17.20	13.95	3.30	1.72	0.00
MSTRG.116	igf1	ENSONIT	GO:0005	04114(Oocyte meiosis);04115(p53 signali	K05459	NA		9.17	10.27	14.07	4.69	6.28	4.67	2.01	1.00	0.02
MSTRG.118	smc1al	ENSONIT	GO:0000	04110(Cell cycle);04111(Cell cycle - ye	K06636	NA		4.03	4.16	3.67	0.81	1.02	1.42	2.32	1.21	0.00
MSTRG.162	camk2d2	MSTRG.1	NA	04012(ErbB signaling pathway);04020(Cal	K04515	EC:2.7.11.17		0.64	1.74	0.29	3.70	4.02	4.16	0.32	-1.65	0.00
MSTRG.105	IL1RAP	MSTRG.1	GO:0006	04010(MAPK signaling pathway);04020(Cal	K06268	NA		13.00	19.58	14.05	45.42	59.56	37.65	0.75	-0.41	0.01
MSTRG.113	AKT1	MSTRG.1	GO:0005	04010(MAPK signaling pathway);04012(Erb	K04456	EC:2.7.11.1		24.12	19.47	19.85	4.44	5.66	11.98	1.10	0.13	0.05
MSTRG.117	mapk14a	ENSONIT	GO:0005	04010(MAPK signaling pathway);04011(MAP	K04441	EC:2.7.11.24		10.81	12.27	10.42	21.90	22.99	32.43	0.88	-0.18	0.04
MSTRG.126	akt2	ENSONIT	GO:0005	04010(MAPK signaling pathway);04012(Erb	K04456	EC:2.7.11.1		10.82	11.18	9.75	4.83	5.26	3.14	1.09	0.13	0.04
MSTRG.164	PPP3CA	ENSONIT	GO:0005	04010(MAPK signaling pathway);04020(Cal	K04348	EC:3.1.3.16		4.99	3.47	3.59	12.90	12.64	11.60	0.92	-0.13	0.01
MSTRG.174	rps6ka1	MSTRG.1	GO:0005	04010(MAPK signaling pathway);04114(Ooc	K04373	EC:2.7.11.1		2.63	4.63	4.50	10.89	9.70	11.34	0.97	-0.05	0.03
MSTRG.178	ppp3ccb	MSTRG.1	GO:0005	04010(MAPK signaling pathway);04020(Cal	K04348	EC:3.1.3.16		6.97	6.95	8.10	2.64	2.86	2.15	1.08	0.12	0.04
MSTRG.180	raf1b	MSTRG.1	GO:0005	04010(MAPK signaling pathway);04012(Erb	K04366	EC:2.7.11.1		3.56	5.65	4.73	9.44	12.57	14.12	1.19	-0.23	0.04
MSTRG.198	mapk9	MSTRG.1	GO:0044	04010(MAPK signaling pathway);04012(Erb	K04440	EC:2.7.11.24		2.57	2.44	3.17	9.62	6.84	5.29	1.28	-0.14	0.02
MSTRG.205	rps6kal	ENSONIT	GO:0005	04010(MAPK signaling pathway);04114(Ooc	K04373	EC:2.7.11.1		8.81	1.84	4.66	24.28	15.36	13.51	1.27	-0.35	0.02
MSTRG.325	kras	ENSONIT	GO:0005	04010(MAPK signaling pathway);04012(Erb	K07827	NA		6.22	7.00	6.05	1.79	3.58	2.07	0.85	0.25	0.02
MSTRG.396	mapk8a	ENSONIT	GO:0005	04010(MAPK signaling pathway);04012(Erb	K04440	EC:2.7.11.24		10.57	13.00	9.83	3.32	3.10	3.26	0.91	0.36	0.01
MSTRG.447	MAPK14 (1 c	ENSONIT	GO:0005	04010(MAPK signaling pathway);04011(MAP	K04441	EC:2.7.11.24		14.76	9.52	11.84	7.55	5.40	5.02	0.78	0.34	0.03
MSTRG.462	AKT2 (1 of	ENSONIT	GO:0005	04010(MAPK signaling pathway);04012(Erb	K04456	EC:2.7.11.1		9.80	17.25	15.37	5.90	4.29	6.26	1.03	0.05	0.04
MSTRG.519	prkacaa	MSTRG.5	GO:0005	04010(MAPK signaling pathway);04020(Cal	K04345	EC:2.7.11.11		14.01	9.88	8.46	1.67	2.84	2.07	1.37	0.46	0.02
MSTRG.554	prkacbb	ENSONIT	NA	04010(MAPK signaling pathway);04020(Cal	K04345	EC:2.7.11.11		5.03	5.33	2.98	5.00	5.23	5.26	0.83	-0.27	0.03
MSTRG.138	cox6b1	MSTRG.1	GO:0004	00190(Oxidative phosphorylation);04260(	K02267	NA		91.75	106.36	110.15	34.38	24.00	56.19	2.56	1.36	0.03
MSTRG.151	eno3	ENSONIT	GO:0005	00010(Glycolysis / Gluconeogenesis);030	K01689	EC:4.2.1.11		554.25	444.70	752.39	1816.13	1387.31	892.11	0.44	-1.19	0.03
MSTRG.231	gpib	ENSONIT	GO:0005	00010(Glycolysis / Gluconeogenesis);000	K01810	EC:5.3.1.9		44.97	46.04	76.91	223.46	116.28	112.17	0.44	-1.19	0.01
MSTRG.434	pfkmb	MSTRG.4	GO:0003	00010(Glycolysis / Gluconeogenesis);000	K00850	EC:2.7.1.11		262.08	346.55	354.52	731.61	746.06	598.65	0.46	-1.12	0.01
MSTRG.162	camk2d2	MSTRG.1	NA	04012(ErbB signaling pathway);04020(Cal	K04515	EC:2.7.11.17		0.64	1.74	0.29	3.70	4.02	4.16	0.32	-1.65	0.00
MSTRG.100	cox7a2a	ENSONIT	GO:0005	00190(Oxidative phosphorylation);04260(	K02270	NA		13.56	11.30	13.93	34.39	30.83	34.96	0.74	-0.43	0.00
MSTRG.102	ATP6V1A (1	ENSONIT	GO:0005	00190(Oxidative phosphorylation);04145(	K02145	EC:3.6.3.14		2.73	1.54	1.91	4.20	4.74	6.39	0.89	-0.17	0.04
MSTRG.105	ndufa9a	ENSONIT	GO:0005	00190(Oxidative phosphorylation);05010(	K03953	NA		31.34	35.90	27.75	8.62	7.13	14.53	1.14	0.20	0.02
MSTRG.134	uqcrc2b	MSTRG.1	GO:0005	00190(Oxidative phosphorylation);04260(	K00415	NA		108.97	113.35	92.17	54.11	58.06	52.65	1.86	0.90	0.00
MSTRG.137	ATP5H (1 of	ENSONIT	GO:0000	00190(Oxidative phosphorylation);05010(	K02138	NA		204.95	265.19	193.05	92.54	77.83	97.69	1.04	0.06	0.01
MSTRG.138	ndufb7	MSTRG.1	GO:0005	00190(Oxidative phosphorylation);05010(	K03963	NA		23.73	26.61	18.12	10.29	9.35	6.90	1.16	0.21	0.04
MSTRG.138	cox6b1	MSTRG.1	GO:0004	00190(Oxidative phosphorylation);04260(	K02267	NA		91.75	106.36	110.15	34.38	24.00	56.19	2.56	1.36	0.03
MSTRG.140	ndufc2	MSTRG.1	GO:0016	00190(Oxidative phosphorylation);05010(	K03968	NA		11.09	13.97	16.26	23.93	22.69	48.54	0.91	-0.13	0.03
MSTRG.117	adcy6b	ENSONIT	GO:0016	00230(Purine metabolism);04062(Chemokir	K08046	EC:4.6.1.1		2.19	2.51	0.93	5.67	7.69	7.74	0.88	-0.18	0.03
MSTRG.118	cacna1sb	ENSONIT	GO:0005	04010(MAPK signaling pathway);04020(Cal	K04857	NA		98.07	76.81	87.82	141.06	136.41	117.70	0.70	-0.51	0.00
MSTRG.252	mmp14a	MSTRG.2	GO:0031	04912(GnRH signaling pathway)	K07763	EC:3.4.24.80		4.39	7.68	3.95	12.97	11.00	11.40	0.61	-0.72	0.20
MSTRG.372	calm3a	MSTRG.3	GO:0005	04020(Calcium signaling pathway);04070(	K02183	NA		71.28	157.43	104.45	25.47	24.45	35.43	1.28	0.35	0.36
MSTRG.158	casp3b	MSTRG.1	NA	04010(MAPK signaling pathway);04115(p53	K02187	EC:3.4.22.56		3.10	5.75	5.70	1.56	2.91	1.70	1.00	0.00	0.04
